# *Drosophila suzukii* preferentially lays eggs on spherical surfaces with a smaller radius

**DOI:** 10.1038/s41598-022-20022-z

**Published:** 2022-09-22

**Authors:** Junichi Akutsu, Takashi Matsuo

**Affiliations:** Laboratory of Applied Entomology, Department of Agricultural and Environmental Biology, The University of Tokyo, 1-1-1 Yayoi, Bunkyo-ku, Tokyo Japan

**Keywords:** Agroecology, Behavioural ecology, Invasive species

## Abstract

*Drosophila suzukii* is an agricultural pest that predominantly harms small fruits, having a serrated ovipositor that is able to pierce the skin of ripening fruits. Its oviposition preference has been studied from various aspects including chemical and physical properties of oviposition substrates. However, its preference for certain shapes or sizes of substrates has not been explored. In this study, we tested the oviposition preference of *D. suzukii* for artificial oviposition substrates with different surface curvatures using 27 strains recently established from wild populations collected in Japan. We found that *D. suzukii* laid more eggs on a surface with smaller radii (4.8 and 5.7 mm) compared with larger radii (7.7 and 9.6 mm). We also found that the most preferred radius differed among strains. Notably, the preference was independent of the volume of substrates, suggesting that *D. suzukii* uses the surface curvature as a cue for its oviposition site selection. These results provide an additional explanation for why *D. suzukii* preferentially uses small fruits as its oviposition sites.

## Introduction

*Drosophila suzukii*, the spotted wing Drosophila, is an invasive agricultural pest expanding its range around the world^1,2^. *D. suzukii* has a serrated ovipositor that is able to penetrate the skin of ripening fruits and preferentially damages small fruits^3,4^. *D. suzukii* uses various cues in its oviposition site selection, including chemical cues (odorants, tastants, and acidity) and physical cues (colour, firmness, and texture)^4–23^. Among them, firmness of the fruit skin has been considered to play an important role in limiting the host range of *D. suzukii* to small fruits^2^. In agreement with the morphological characteristic in its ovipositor, *D. suzukii* could lay eggs on relatively harder artificial substrates compared with other Drosophila species^16^. In contrast, the much harder skin of large fruits has been thought to prevent oviposition even by *D. suzukii*, limiting its hosts to small fruits^1–3^. Although firmness of the fruit skin explains why *D. suzukii* cannot harm large fruits, it does not exclude the possibility that *D. suzukii* prefers small fruits for other reasons. In fact, its oviposition preference for certain shapes or sizes of substrates has not been tested experimentally.

In preliminary experiments using artificial oviposition substrates, we accidentally found that *D. suzukii* laid more eggs on spherical surfaces than on flat surfaces. In this study, we examined whether *D. suzukii* prefers a certain surface curvature, using multiple strains collected in Japan. We also tried to disentangle the effects of substrate size and surface curvature controlling the volume of substrates independently from the curvature.

## Material and methods

### *D. suzukii* strains

*D. suzukii* strains were established from wild populations collected at various locations in Japan during 2019–2021 (Table 1). In most cases, single pairs that emerged from collected host fruits were used to establish the strains to minimize the influence of possible genetic drift during the laboratory culture by reducing the initial genetic variation within a strain. Some strains were established using other methods (trap collection as a source, and a single mated female or multiple pairs as an origin). The strains were cultured in the same way as for *D. melanogaster*. Newly eclosed adults were transferred into vials containing standard Drosophila culture medium made of corn meal and glucose and maintained at 20 °C with the 16L:8D light cycle until the next generation emerged. In this condition, one generation took 3 weeks (17 generations/year). Individuals used for experiments were reared at 25 °C from the larval stage.

**Table 1:** List of strains.

ID	Location	Date of collection	Host	Origin
#1	Hitotsuya, Adachi, Tokyo	May, 2020	*Cerasus jamasakura*	Single pair
#2	Hokima, Adachi, Tokyo	May, 2021	*Cerasus jamasakura*	Single pair
#3	Hitotsuya, Adachi, Tokyo	May, 2021	*Cerasus jamasakura*	Single pair
#4	Mizumoto Koen, Katsushika, Tokyo	June, 2020	Mulberry	Single pair
#5	Kitanomaru Koen, Chiyoda, Tokyo	September, 2021	Chinese dogwood	Single pair
#6	Oji, Kita, Tokyo	May, 2021	*Cerasus jamasakura*	Single pair
#7	Yayoi, Bunkyo, Tokyo	May, 2021	*Cerasus jamasakura*	Single pair
#8	Yayoi, Bunkyo, Tokyo	May, 2021	*Cerasus jamasakura*	Single pair
#9	Midori, Chiba, Chiba	September, 2021	Chinese dogwood	Single pair
#10	Midori, Chiba, Chiba	July, 2021	Blueberry	Single pair
#11	Naka, Nagareyama, Chiba	October, 2020	American pokeweed	Single pair
#12	Furumagi, Nagareyama, Chiba	October, 2020	American pokeweed	Single pair
#13	Omoi, Nagareyama, Chiba	October, 2020	Trap	Single pair
#14	Nagasaki, Nagareyama, Chiba	October, 2020	Trap	Single pair
#15	Namiki, Tokorozawa, Saitama	September, 2021	Chinese dogwood	Single pair
#16	Namiki, Tokorozawa, Saitama	September, 2021	American pokeweed	Single pair
#17	Funako, Atsugi, Kanagawa	May, 2019	Mulberry	Multiple pairs
#18	Funako, Atsugi, Kanagawa	October, 2019	Trap	Single pair
#19	Nuda, Ashigara, Kanagawa	September, 2021	Chinese dogwood	Single pair
#20	Kuno, Odawara, Kanagawa	September, 2021	Chinese dogwood	Single pair
#21	Kounan, Yokoyama, Kanagawa	September, 2021	Chinese dogwood	Single pair
#22	Amakubo, Tsukuba, Ibaraki	September, 2021	Chinese dogwood	Single pair
#23	Ouchihikami, Yamaguchi, Yamaguchi	July, 2021	Blueberry	Single pair
#24	Tanaka, Kuroishi, Aomori	2015	Blueberry	Unknown
#25	Minorigaoka, Yamagata, Yamagata	September, 2021	Chinese dogwood	Single pair
#26	Kashiwa, Kashiwa, Chiba	May, 2021	Mulberry	Multiple pairs
#27	Omiya, Chichibu, Saitama	June, 2021	*Cerasus jamasakura*	Multiple pairs

### Four-choice assay

**Figure 1:**
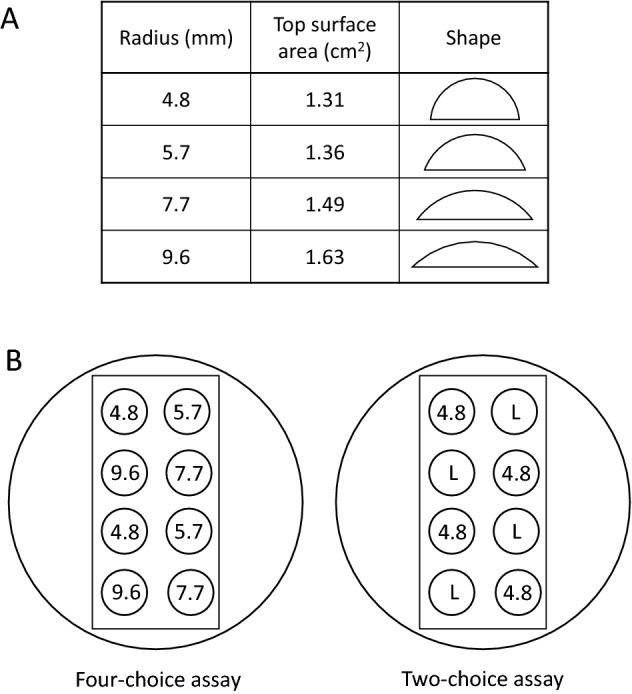
Schematic representation of the experimental setups. (**A**) Shape of the oviposition substrates. (**B**) Arrangement of the oviposition substrates in the oviposition assay. In a disposable petri dish, a piece of cotton pad was placed and soaked with water. Eight oviposition substrates were put on the cotton pad. “L” in two-choice assay designates oviposition substrates with either of 5.7, 7.7, or 9.6 mm radius.

Oviposition substrate was made of 2% agar solution (Seakem® LE Agarose, Lonza, Basel, Switzerland) cast in silicone moulds for UV resin-crafting hobbies, which have hemispherical holes of various sizes. Oviposition substrates with different surface curvatures of the same volume were prepared by pouring 200 μl of agar solution into each hole and allowed to solidify (shapes of the oviposition substrates are shown in Fig. 1A). The tested range of the curvature radius (4.8–9.6 mm) was determined considering the natural hosts of *D. suzukii* such as raspberry and wild cherry. Ten females at the age of 7 to 9 days after eclosion were introduced into a petri dish (9 cm diameter), in which 8 oviposition substrates were placed on a wet cotton pad (3 × 6 cm, Fig. 1B). The assay started 6 h before the transition to the dark phase. After 24 h of oviposition at 25 °C, the number of eggs laid on each substrate was counted. Each fly was used only once. Ten replications were made for each strain. When the total number of eggs in a dish was less than 10, the corresponding data were excluded from the analysis, and additional replications were made.

### Two-choice assay

Four *D. suzukii* strains that showed different strength of preference for the smallest radius in the four-choice assay were selected for a two-choice assay (strain #17: stronger preference, #20: weaker preference, #22 and #26: moderate preference). *D. melanogaster* (CS) was used for comparison. Flies were prepared in the same way as the four-choice assay. Each dish contained two sizes of substrates, among which smaller ones were fixed to a 4.8 mm radius and the others were one of 5.7, 7.7, and 9.6 mm (Fig. 1B). Ten replications were made for each size pair. To test the cross-modal effect between taste and curvature, the same assay was repeated using the substrates containing 1% (555 mM) glucose using independent individuals. Preference index was calculated as (Number of eggs laid on smaller substrates − Number of eggs laid on larger substrates) ÷ Total egg number.

### Statistical analysis

All the data were analysed using R version 4.2.0^24^. The result of four-choice assay was analysed by fitting two models. Model 1 was for examining the effect of the curvature radius and strains on the number of eggs. The response variable of the model was the number of eggs laid on the substrates with a focal radius, assumed to have the Poisson error distribution. The explanatory variables were radius (fixed effect: continuous) and strains (random effect: categorical). To compare the effect size with that of strains, the values for the radius were transformed as 4.8 mm: 0; 5.7 mm: 1; 7.7 mm: 2; and 9.6 mm: 3 (continuous). The parameters were estimated using the glmmML package with the Gauss–Hermite method^25^. Model 2 was used for examining the difference between strains in the tendency to prefer the smallest radius. The response variable was the pairs of egg numbers on the smallest (4.8 mm) substrates vs. those on the other three sizes (binomial error distribution). The explanatory variable was strain (fixed effect: categorical). The parameters were estimated using the glm function included in the R base distribution. The results of the two-choice assays were analysed by fitting Model 3, in which the response variable was the pairs of egg numbers on the smaller (4.8 mm) substrates vs. those on the larger ones (binomial error distribution), and the explanatory variables were strain (categorical), glucose (categorical), radius (continuous), and interactions between them. The value for radius was transformed as follows for comparisons of the effect size with other categorical factors; 5.7 mm vs. 4.8 mm: 0; 7.7 mm vs. 4.8 mm: 1; 9.6 mm: vs. 4. 8 mm: 2. The parameters were estimated using the glm function. In all the models, default link functions were used.

### Ethical approval

*D. suzukii* was collected in areas where it was allowed. No licences or permits were required for this research in Japan. Nevertheless, we adhered to the ASAB/ABS Guidelines for use and disposal of the animals in this study.

## Results

### Four-choice assay

**Figure 2:**
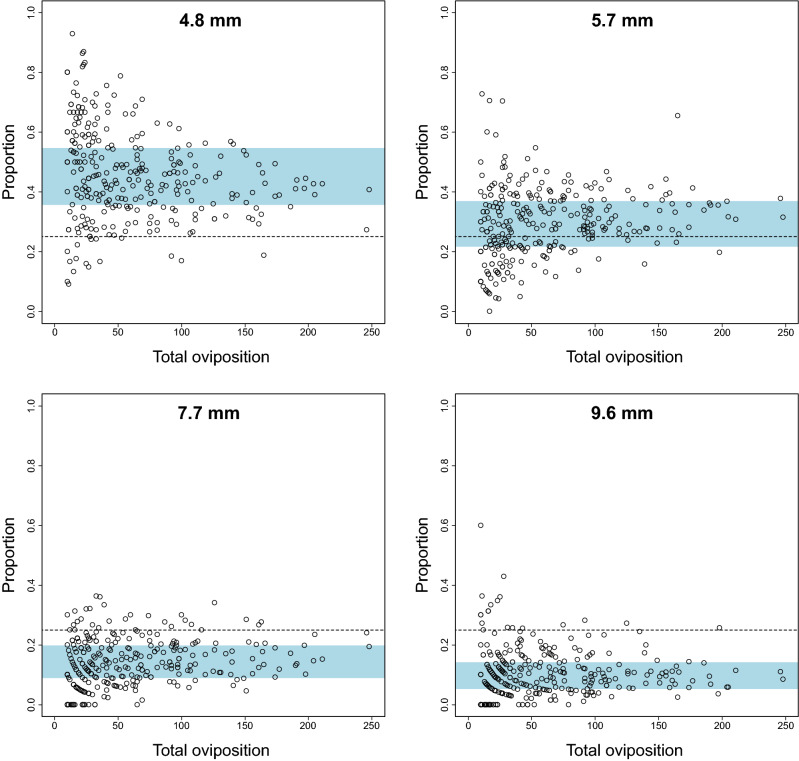
Results of the four-choice assay. The proportion of the number of eggs on each size of substrates to the total egg number on all substrates was plotted. Each point represents a single replicate. Ten replicates were made for each of the 27 strains, in total 270 times of assay were conducted. Dotted lines indicate the proportion of 0.25, the expected value of the null hypothesis (no preference). Shading represents the range of proportion between the upper and lower quartiles that contains 50% of data.

**Figure 3:**
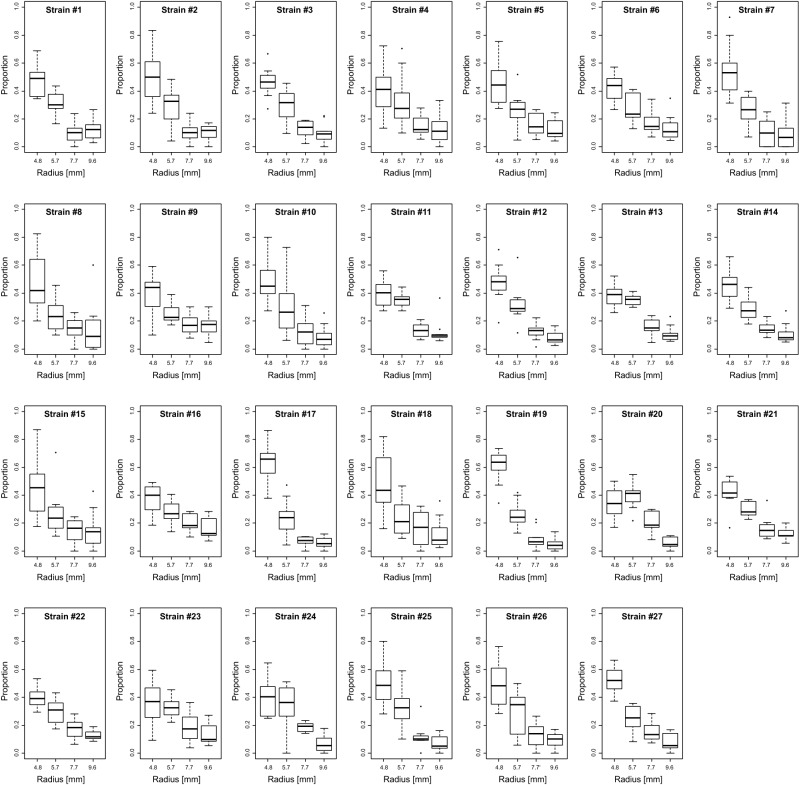
Strain-wise plot of the results of the four-choice assay. Proportion of eggs laid on each size of the oviposition substrate to the total egg number is shown. The assay was repeated for 10 times for each strain.

The total number of eggs varied considerably between replicates (Fig. 2). The effect of strains on the total egg number was statistically significant (Table 2 Model 1). The proportion of eggs laid on each size of substrate to the total egg number also varied particularly when the total egg number was small. However, the tendency of preference for smaller substrates was obvious and statistically significant (Fig. 2, Table 2 Model 1). Comparing between the strains, some strains showed a stronger preference for the smallest substrates (strains #10, #17, #19) or a weaker preference (#13, #16, #20, #24) compared with the other strains (Fig. 3, Table 2 Model 2).

**Table 2:** Analysis of the result of four-choice assay.

Explanatory variables	Estimated coefficient	Standard error	z (Wäld test)	P value
Model 1 (GLMM): Response variable = Number of eggs (Poisson)				
(Intercept)	3.2122	0.10613	30.27	0
Radius (fixed)*	−0.4822	0.007256	−66.46	0
Strain (random)	0.5487**	0.07521	NA***	0
Model 2 (GLM): Response variable = Number of eggs laid on 4.8 mm vs. the others (binomial)				
(Intercept)****	−0.24088	0.08336	−2.890	0.003857
Strain #2	0.23410	0.14321	1.635	0.102109
Strain #3	0.03609	0.11332	0.318	0.750124
Strain #4	−0.03983	0.15232	−0.261	0.793719
Strain #5	−0.02506	0.11524	−0.217	0.827866
Strain #6	−0.07483	0.11284	−0.663	0.507233
Strain #7	0.29804	0.16128	1.848	0.064611
Strain #8	−0.02453	0.12138	−0.202	0.839873
Strain #9	−0.07553	0.12125	−0.623	0.533334
**Strain #10**	**0.24936**	**0.12419**	**2.008**	**0.044661**
Strain #11	−0.16271	0.10033	−1.622	0.104868
Strain #12	−0.03195	0.10537	−0.303	0.761742
**Strain #13**	**−0.25969**	**0.09839**	**−2.639**	**0.008307**
Strain #14	0.01008	0.10612	0.095	0.924343
Strain #15	0.06460	0.13425	0.481	0.630361
**Strain #16**	**−0.20279**	**0.10100**	**−2.008**	**0.044656**
**Strain #17**	**0.65005**	**0.12726**	**5.108**	**0.000000**
Strain #18	−0.11620	0.11760	−0.988	0.323139
**Strain #19**	**0.52639**	**0.12069**	**4.362**	**0.000013**
**Strain #20**	**−0.41886**	**0.11174**	**−3.749**	**0.000178**
Strain #21	−0.02134	0.10063	−0.212	0.832035
Strain #22	−0.17619	0.11578	−1.522	0.128065
Strain #23	−0.17397	0.12158	−1.431	0.152450
**Strain #24**	**−0.29452**	**0.12056**	**−2.443**	**0.014570**
Strain #25	0.02750	0.12540	0.219	0.826438
Strain #26	0.07128	0.12525	0.569	0.569291
Strain #27	0.18102	0.11513	1.572	0.115859

Significant values are in bold.

* Values for the radius was transformed as {4.8 mm: 0; 5.7 mm; 1, 7.7 mm: 2; 9.6 mm: 3} (continuous).

**For the random effect, estimated standard deviation is shown.

***For the random effect, *P* value was calculated using the bootstrap test.

****Strain #1 was assigned as a base strain (Intercept represents the preference of strain #1).

### Two-choice assay

**Figure 4:**
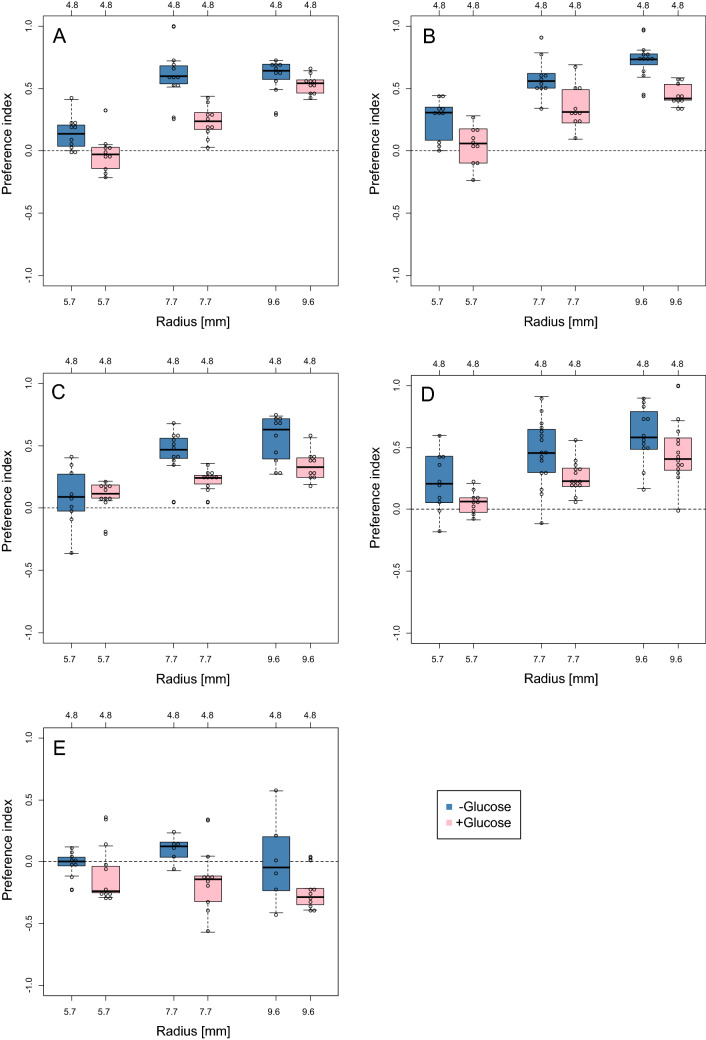
Results of the two-choice assay. (**A**–**D**) *D. suzukii* strains #17, #20, #22, and #26, respectively. (**E**) *D. melanogaster* CS. The x axis indicates the radius of the larger substrates. The smaller substrates were fixed as 4.8 mm. Preference index = (Number of eggs laid on smaller substrates − Number of eggs laid on larger substrates) ÷ Total egg number. Positive values of the preference index indicate the preference for a smaller radius.

*D. suzukii* showed a preference for substrates with a smaller radius, whereas *D. melanogaster* did not (Fig. 4, Table 3). Addition of glucose to the substrates weakened the preference in *D. suzukii*, whereas it potentiated the preference for larger substrates in *D. melanogaster*. The effect of interaction between strain and radius was significant and positive for *D. suzukii* strain #17 (stronger preference), suggesting that this strain was more sensitive to the increasing difference in the surface curvature (Table 3).

**Table 3:** Analysis of the result of two-choice assay.

Explanatory variables	Estimated coefficient	Standard error	z (Wäld test)	P value
Model 3 (GLM): Response variable = Number of eggs laid on 4.8 mm vs. larger substrates (binomial)				
(Intercept)*	0.40797	0.08242	4.950	0.000001
**Strain**				
Strain #17	−0.06673	0.09747	−0.685	0.493568
Strain #20	0.15152	0.09494	1.596	0.110477
Strain #22	0.01885	0.09550	0.197	0.843523
***D. melanogaster* (CS)**	**−0.40269**	**0.11760**	**−3.424**	**0.000617**
**Glucose + **	**−0.29578**	**0.08449**	**−3.501**	**0.000464**
Radius**	**0.53651**	**0.05430**	**9.881**	**0.000000**
**Strain:Glucose + **				
#17:Glucose +	−0.06380	0.09819	−0.650	0.515849
#20:Glucose +	−0.11015	0.09836	−1.120	0.262802
#22:Glucose +	0.02884	0.09610	0.300	0.764129
mel:Glucose +	−0.02628	0.11661	−0.225	0.821672
**Strain:radius**				
**#17:radius**	**0.12281**	**0.06022**	**2.039**	**0.041407**
#20:radius	0.03457	0.05994	0.577	0.564115
#22:radius	−0.11092	0.05713	−1.941	0.052209
**mel:radius**	**−0.50909**	**0.06280**	**−8.107**	**0.000000**
**Glucose + :radius**	**−0.13396**	**0.03938**	**−3.402**	**0.000669**

Significant values are in bold.

*Strain #26 was assigned as a base strain (Intercept represents the preference of strain #26 in 5.7 vs. 4.8 without glucose).

**Values for the radius was transformed as {5.7 mm vs.4.8 mm: 0; 7.7 mm vs. 4.8 mm: 1; 9.6 mm: vs. 4.8 mm: 2} (continuous).

## Discussion

Our results showed that *D. suzukii* preferentially laid eggs on spherical surfaces with a smaller radius. Because all oviposition substrates were provided in the same volume and with comparable surface area, *D. suzukii* should use curvature as a direct cue of this preference. This finding provides additional explanation for the preferential use of small fruits as the oviposition sites in this species. Surprisingly, this characteristic of *D. suzukii* has been overlooked until now. There are three possibilities for this.

First, the preference for curvature may be weak compared with the preferences for other cues. Our results showed that the addition of glucose to the substrate reduced the preference for curvature (Fig. 4). If other oviposition cues such as odours, tastes, and firmness are provided appropriately, *D. suzukii* may lay eggs regardless of the substrate curvature. Nevertheless, it would be noteworthy that our preliminary competitive cross-modal experiments indicated that the preference for curvature overrides at least those for glucose and firmness (data not shown). Further experiments with various other cues will prove the significance of curvature preference in the *D. suzukii* oviposition behaviour.

Second, there may be an unexpected bias when establishing and maintaining laboratory stocks. Nearly all laboratories use the culture medium that has a flat surface, which may select against strains maintaining a strict preference for curvature. In fact, our results showed that some strains have a different preference for curvature, suggesting that natural variation exists as a potential target of such selection. Furthermore, strains retaining a preference for curvature may lay few eggs on flat surfaces, which should be an unfavourable characteristic to be used as a “standard” strain in oviposition experiments. We tried to avoid this type of unintended selection and bias by reducing the genetic variation within a strain, as well as by using relatively newly established strains. Even so, we experienced that some strains were quite difficult to propagate sufficiently for experimental use. In contrast, strain #24 was exceptional in this regard because it has been maintained for 6 years in a laboratory. As expected, it showed a weaker preference to the smallest radius (Fig. 3, Table 2 Model 2).

Third, our results may reflect the difference between wild populations in the native range and the invaded area. Plums and strawberries are larger than raspberry and blueberry and have been reported as major agricultural products damaged by *D. suzukii* in invaded areas^3,26,27^, whereas they are not seriously damaged in Japan compared with smaller fruits, indicating that the preference for curvature has been already lost or weakened to a certain extent in the wild populations of the invaded area. *D. suzukii* is thought to adapt to various environments quickly^1^. Loss of preference to curvature may have occurred as a part of adaptation to new environments.

The above possibilities are not exclusive to each other. Among them, the last one should be most important from the biological point of view because it is related to the ecological significance of this preference—what is the selection pressure maintaining it in the native range? There are several possibilities. (1) *D. suzukii* is inferior to other Drosophila species in competition during the larval stage. If inter-species competition is more severe on larger fruits, preferential oviposition on smaller fruits will be selected. (2) Because ripening fruit is more likely to be foraged than rotting fruits by birds and other vertebrates, predation risk on larger fruits may select the preference for smaller fruits only in *D. suzukii*. (3) Aggregate fruit (raspberry and blackberry) and collective fruit (mulberry) have higher surface curvature than simple fruit of the same size, and *D. suzukii* may use the curvature as a cue of these hosts. Nevertheless, it should be noted here that our results do not exclude the possibility that the preference for spherical surfaces is a trait shared with other Drosophila species in the wild, which may have been lost from most laboratory strains. Further studies are required to examine these possibilities.

Since the pioneering work by Ishii (1952) reporting that the adzuki bean beetle, *Callosobruchus chinensis*, preferentially laid eggs on smaller glass beads^28^, the effect of substrate size on oviposition behaviour has been studied in a few groups of insects. Preferential oviposition on certain sizes of substrates was reported in the boll weevil *Anthonomus grandis grandis* and three “true” fruit fly species^29–33^. Parasitoid wasps control offspring sex ratio depending on the host size^34^. Experiments using artificial substrates showed that curvature rather than surface area influenced oviposition of the adzuki bean beetle and the Indianmeal moth *Plodia interpunctella*^35,36^. A mechanism of host-radius measurement was proposed in the parasitoid wasp *Trichogramma minutum*^37^. In *D. melanogaster*, sexually dimorphic mechanosensory neurons on the terminal segments were shown to regulate copulation duration^38^. However, no mechanism for curvature sensation has been reported so far, probably because the laboratory strains of *D. melanogaster* do not show any preference for the curvature of oviposition substrates. In *D. suzukii*, several lines of evidence support the existence of mechanosensory mechanisms on the ovipositor, although their involvement in the curvature sensation has not been examined^10,39^. Considering that our knowledge in this field is limited, *D. suzukii* may serve as an important model system to study the mechanism underlying the perception of surface curvature and preference for certain substrate size, as well as its ecological significance.

Besides the biological aspects, our finding is expected to technically contribute to promoting the studies on *D. suzukii* by improving the egg laying rate both in experiments and laboratory cultures. By using spherical oviposition substrates, on which *D. suzukii* readily lays eggs, oviposition behaviour can be examined more precisely and efficiently. Establishing laboratory stocks from wild populations of *D. suzukii* frequently encounters difficulties due to low fecundity^40^. Use of spherical substrates will greatly improve the success rate on such occasions, reducing the loss of precious samples and making the maintenance of laboratory stocks easy. Because it is quite simple to incorporate spherical oviposition substrates into experiments and culture methods, we expect many researchers in this field will be benefited with it.

## Supplementary Information

Supplementary Information.

## Data Availability

The raw data are provided as supplementary information.
